# Epidemiological Characteristics and Survival Analysis of Patients With Nasopharyngeal Cancer in Western Greece

**DOI:** 10.7759/cureus.14711

**Published:** 2021-04-27

**Authors:** Christos S Avdulla, Theodoros Papadas, Nicholas Mastronikolis, Eleni Jelastopulu

**Affiliations:** 1 Department of Public Health, University of Patras, Patras, GRC; 2 Department of Otorhinolaryngology, University of Patras, Patras, GRC

**Keywords:** nasopharyngeal cancer, cohort study, survival analysis, prognostic factors, western greece, tnm staging

## Abstract

Purpose

The purpose of this study is to assess the overall survival (OS) of patients with nasopharyngeal cancer and the factors affecting the survival rates.

Methods

A retrospective cohort study was performed, including 77 patients with nasopharyngeal cancer diagnosed and treated in the Otorhinolaryngology Clinic of the University Hospital of Patras during 1990-2017. The prognostic impact of age, gender, occupation, smoking/alcohol, and TNM staging were evaluated using Kaplan-Meier analysis.

Results

During the last 28 years, nasopharyngeal cancer (NPC) was higher in men (80.5% of patients) than women (19.5%) (mean age 56-years). Most patients were smokers (64.9%, mean 70 pack-years) and 35 (45.5%) of them were alcohol users. Postoperative staging indicated 39% stage-III, 26% stage-IV, and 26% stage-I. Histologically, 70.1% of the volumes were WHO-III, 20.8% WHO-II, and 5.2% WHO-I. Also, 98.7% of patients received radiotherapy, 85.7% chemotherapy, and 20.8% surgery. More than half were farmers (26%), self-employed (16.9%), and workers (14.3). During the follow-up (mean 66 months), 38 (49.5%) patients died, 88.9% from disease-related causes. The 5-year survival was 58.8%, 74.5% for non-smokers, and 49.1% for smokers, and 10-year survival was 43.6%, 63.4%, and 31.6%, respectively (p=0.016). Moreover, significant statistical differences were observed in age (p=0.054), time period of diagnosis and treatment (p=0.002), cause of death (p=0.033), and metastatic disease (p=0.023).

Conclusions

Age, stage in disease detection, tumor characteristics, treatment, and tobacco abuse are important factors that affect the OS of patients with NPC during the three last decades.

## Introduction

Nasopharyngeal carcinoma (NPC) is a relatively rare neoplasm representing 0.8% (1.1% in males and 0.4% in females) of all human neoplasia worldwide [1]. According to global statistics on cancer, in 2018, there were over 129,079 cases of NPC [1]. In addition, one of the peculiarities of this neoplasm is its geographical distribution in the countries of Southeast Asia and the Mediterranean, and consequently, in Greece [1,2].

Nasopharyngeal carcinoma can occur at any age, but the majority of cases are diagnosed in adults between the third and fifth decades. Moreover, symptoms of NPC vary and are atypical in the early stages of the disease, leading to a delay in diagnosis. As a matter of fact, the NPC appears to be associated with smoking, alcohol, dietary, professional, and genetic factors, and histological subtypes that are directly related to the Epstein-Barr virus infection [1,3].

The selected treatment of NPC depends on various factors, and a combination of chemotherapy and radiotherapy is mostly performed, while lymphatic cleansing of the cervix occurs only when the disease is present or relapsed after the initial course of therapy [3]. Furthermore, response to treatment is much more relevant to the histological type of cancer and the presence of distant metastases and less to the extent of topographical disease. Interestingly, the prognosis of the disease in the early stages is very good, while in the most advanced stages, the 5-year survival is limited to 50-70%, remaining higher than many other tumors in the area [4-6].

The aim of this study is to assess the survival (5-year and 10-year) of patients with NPC, the trends of survival during the three last decades, and the factors affecting the survival rates in the University Hospital of Patras, Western Greece.

## Materials and methods

Patients

A retrospective study of a series of patients was carried out which included all patients with cancer diagnosed and treated at the University Hospital of Patras, Western Greece. In the period 1990-2017, 77 patients (55 men and 22 women) were diagnosed with nasopharyngeal cancer. Prognostic factors such as gender, age, occupation, tobacco/alcohol, histology, and staging were assessed using the SPSS (IBM Corp, Armonk, NY, USA) and Kaplan-Meier method.

All patients' data were evaluated for 5-year and 10-year survival rates. The tumors were staged using the 1997 AJCC (American Joint Committee on Cancer) and UICC (Union Internationale Contre le Cancer) criteria. This system takes into account the size of the tumor (T), the number, size, and location of the cervical lymph nodes (N), and the presence or absence of distant metastases (M). Also, at the histological level, the classification for nasopharynx cancer is proposed by the World Health Organization (WHO) and subdivided into three categories.

Ethics

The survey complied with all ethical standards for research. Prior to its inception at the otorhinolaryngology clinic, permission was obtained from the Scientific Council of the University General Hospital of Patras.

Statistical analysis

The collected data were analyzed with the SPSS statistical version v.25 (IBM Corp, Armonk, NY, USA). Data are presented as mean value, standard deviation, range, and percentages. Survival analysis was performed using the Kaplan-Meier method and the comparison between the two survival curves was performed using the log-rank test. The Kaplan-Meier method was selected due to the greater accuracy of the results for small groups and censored data and was used for procuring the 5-year and 10-year survival percentages and graphs. The final point considered in our analysis was overall survival (OS). Time was calculated from the date of diagnosis to the event of interest, which was death due to any cause. Results are considered statistically significant when the p-value is less than or equal to 0.05.

## Results

Demographic results

In the last 28 years, a higher frequency of nasopharyngeal carcinoma (NPC) was observed in males (80.5%) than in females (19.5%) (Table 1). The mean age of the disease was 56 years (range 16-85 years). Most patients were smokers (64.9%) with an average of 70 pack-years (range 0-200 pack-years) and 35 (45.5%) were alcohol users. Specifically, 16.9% light drinkers (they drank less than 1 day per week), 14.3% moderate drinkers (they drank 1 to 3 drinks per day or more times per week), and 14.3% heavy drinker (they drank more than 3 glasses per day). More than 50% of the patients were farmers (26%), self-employed (16.9%), or workers (14.3%).

**Table 1:** Characteristics of the study population

Characteristic	n	%
Gender		
Male	62	80.5
Female	15	19.5
Total	77	100
Smokers		
Yes	50	64.9
No	26	33.8
Total	76	98.7
Alcohol use		
None	40	51.9
Low	13	16.9
Medium	11	14.3
High	11	14,3
Total	75	97.4
Occupation		
Farmers	20	26
Builders	9	11.7
Self- employed	13	16.9
Professional drivers and mechanics	6	7.8
Workers	11	14.3
Others	16	20.8
Total	75	97.4

After diagnosis, 98.7% of the patients received radiotherapy, 85.7% received chemotherapy, while only 16 (20.8%) patients underwent surgery (Table 2). During the observation period, 38 (49.5%) patients died, out of them, 68.4% due to metastatic or generalized carcinomatosis, 18.4% of relapse, and 13.1% of other causes unrelated to the disease. Only three patients (3.9%) had a family history of NPC.

**Table 2:** Clinical characteristics of the study population

Characteristic	n	%
Treatment		
Radio and Chemotherapy	66	85.7
Radiotherapy	10	13
Total	76	98.7
Surgical treatment		
Yes	16	20.8
No	60	77.9
Total	76	98.7
Survival		
Alive	39	50.6
Death	38	49.4
Total	77	100
Cause of death		
Metastasis	26	68,4
Relapse	7	18.4
Other cause	5	13.2
Total	38	100
Family history		
Yes	3	3.9
No	74	96.1
Total	77	100

Post-surgical staging showed most patients being classified in stages-III (39%), IV (26%), and I (26%), while only 7 (9.1%) patients were classified in stage-II (Table 3). Histologically, nasopharyngeal carcinoma based on the World Health Organization (WHO) is subdivided into three categories. Our results showed that 70.1% of the volumes were WHO-III, 20.8% WHO-II, 5.2% WHO-I, and for three patients, no histological report was available.

**Table 3:** Staging and histological characteristics of the study population

Characteristic	n	%
Stage		
I	20	26
II	7	9.1
III	30	39
IV	20	26
T: Size or direct extent of the primary tumour		
Τ 1	61	79.2
Τ 2	10	13
Τ 3	2	2.6
Τ 4	4	5.2
N: Degree of spread to regional lymph nodes		
Ν 0	22	28.6
Ν 1	8	10.4
Ν 2	31	40.3
Ν 3	16	20.8
M: Presence of distant metastasis		
Μ 0	76	98,7
Μ 1	1	1,3
Total	77	100
Histological type		
WHO-I	4	5.2
WHO-II	16	20.8
WHO-III	54	70.1
Total	74	96.1

Survival analysis results

**Figure 1:**
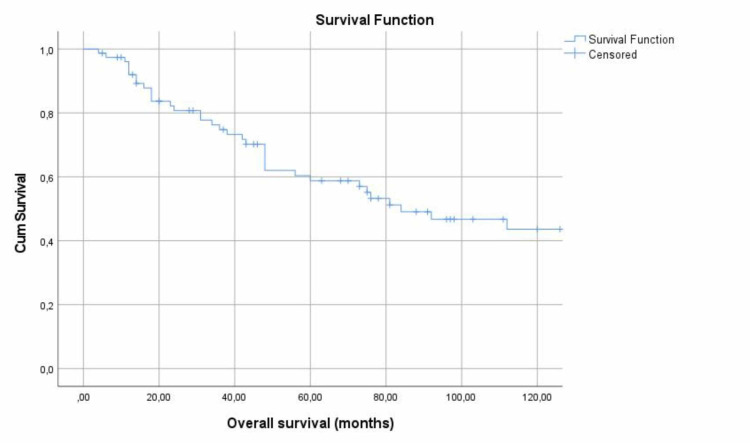
Five-year and 10-year survival of the study population

Five-Year and 10-year survival of the 77 patients with NPC were estimated by Kaplan-Meier analysis. The overall 5- and 10-year survival of NPC patients was 58.8% and 43.6%, respectively (Figure 1).

Based on gender, the results showed that the 5-year survival rate for men was 56.7% and for women 68.9%, whereas the 10-year survival for men (43.2%) did not differ from that for women (43.1%). However, no significant statistical differences were observed between genders (p=0.620) (Table 4).

**Table 4:** Five-year and 10-year overall survival of patients with nasopharyngeal carcinoma according to various demographic and clinical characteristics. P-value between prognostic factors.

Prognostic factors	Overall survival		
	5 year (%)	10 year (%)	p-value
Gender (n=77)			
Male (n=62)	56.7%	43.2%	0.620
Female (n=15)	68.9%.	43.1%	
Age grup (n=77)			
<30 (n=5)	100%	100%	0.054
30-50 (n=22)	76%	60.8%	
51 έως 70 (n=38)	49.8%	34.9%	
>70 (n=12)	45.5%	27.3%	
Stage (ΤΝΜ) (n=77)			
Stage-I (n=20)	73.2%	43.9%	0.179
Stage-II (n=7)	71.4%	47.6%	
Stage-III (n=30)	40.3%	26.8%	
Stage-IV (n=20)	76.5%	66.9%	
Metastasis (ΤΝΜ) (n=77)			
M0 (n=76)	59.6%	44.2%	0.007
M1 (n=1)	0%		
Smokers (n=76)			
Yes (n=50)	49.1%	31.6%	0.016
No (n=26)	74.5%	63.4%	
Histological type (n=74)			
WHO-I (n=4)	33.3%	33.3%	0.945
WHO-II (n=16)	56.3%	35.7%	
WHO-III (n=54)	58.8%	47.5%	
Treatment period (n=76)			
Radio-Chemotherapy (n=66)	64.1%	46.1%	0.53
Radiotherapy (n=10)	23.3%	23.3%	
Surgical treatment (n=76)			
Yes (n=16)	58.2%	23.3%	0.673
No (n= 60)	58.5%	47.8%	
Cause of death			
Metastasis	32%	12%	0.033
Relapse	0%		
Another cause	50%	0%	
Treatment period (n=76)			
1990-1996	14.3%	--	0.002
1997-2003	30.8%	15.4%	
2004-2010	70%	53.8%	
2011-2017	92.3%	(---)	

**Figure 2:**
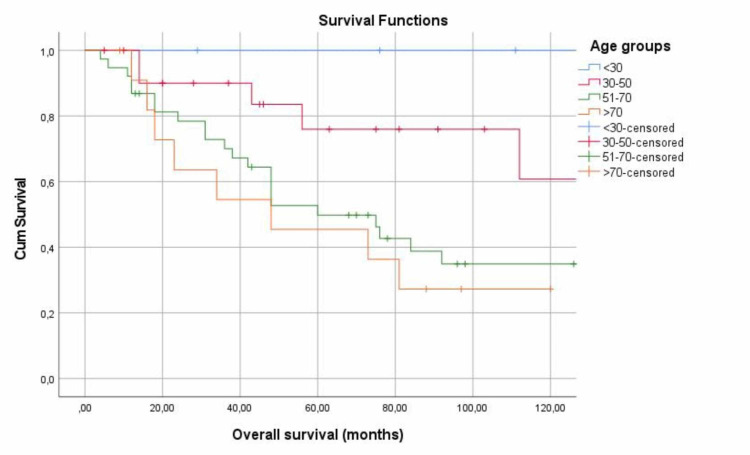
5-year and 10-year survival of age groups

Subsequently, the patients were categorized based on their age (p=0.054). Specifically, in patients younger than 30 years, both, the 5-year and 10-year survival was 100%, while among those aged 30-50 years, the survival reached 76% and 60.8%, respectively. For patients aged 51 to 70 years, 5- and 10-year survival was 49.8% and 34.9% respectively, whereas in patients over 70 years old, the 5- and 10-year survival was calculated at 45.5% and 27.3%, respectively (Figure 2).

Based on Metastases (M), Kaplan-Meier analysis results showed that patients with the non-metastatic disease (M0) had a 5-year survival of 59% and 10-year survival of 45.2%. In contrast, when the tumor has spread to areas of the body away from the nasopharynx (M1), 5-year survival was 0% (p=0.007) (Table 4). 

The staging of nasopharyngeal carcinoma was based on the TNM Staging System. For stage-I, the 5-year survival rate was 73.2%, whereas the 10-year survival was 43.9%, for stage-II, 71.4% and 47.6%, and for stage-III, 40.3% and 26.8%, respectively. However, for stage-IV, the 5-year survival rate was 76.5%, and after 10 years, it was still 66.9% (Table 4).

Histologically and based on the World Health Organization (p=0.945), Kaplan-Meier analysis showed that in squamous cell carcinoma (WHO type I) both 5-year and 10-year survival rates were 33.3%. In non-coronary squamous cell carcinoma (WHO type II), the 5-year survival was 56.3% and 10-year survival 35.7%. Moreover, unlike the two previous types, in undifferentiated carcinoma/lymphoepithelioma (WHO type III), 5-year survival was 58.8%, whereas 10 years later, 47.5% of the patients were still alive (Table 4).

**Figure 3:**
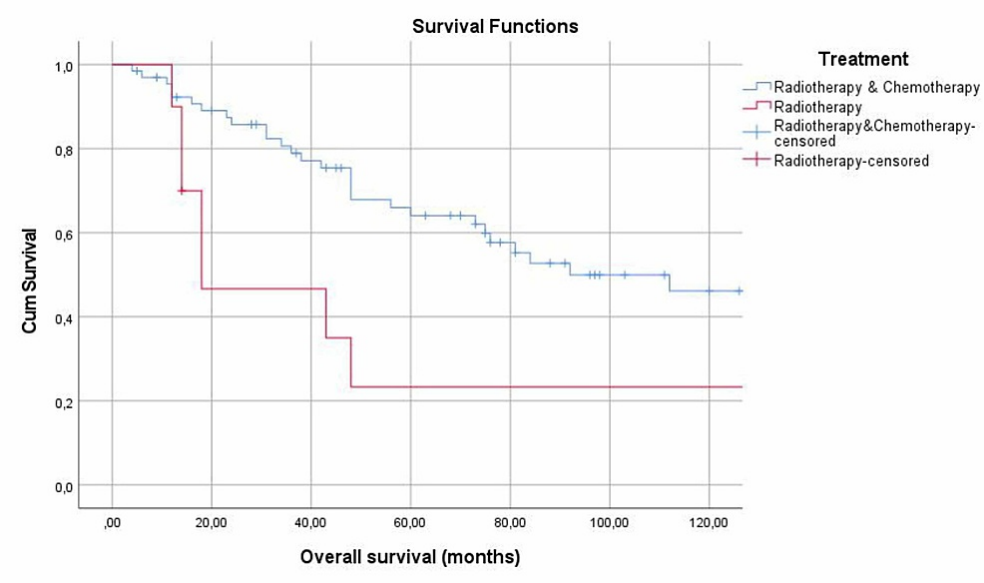
5- and 10-year survival in association to treatment

Because of the anatomical location of NPC and its tendency for rapid bilateral spread to the cervical lymph nodes, the selected treatment is mainly radiotherapy alone or in combination with chemotherapy. Patients undergoing chemotherapy and radiotherapy were analyzed during the course of the disease, and their 5- and 10-year survival rates were 64.1% and 46.1%, respectively. In contrast, patients who receive only radiotherapy (p=0.05) had a 5-year and 10-year survival of 23.3% (Figure 3). Furthermore, the 5- and 10-year survival of patients undergoing surgery was 58.2% and 23.3%, whereas in patients who did not enter surgery (p=0.673), the rate was 58.5% and 47.8%, respectively (Table 4).

**Figure 4:**
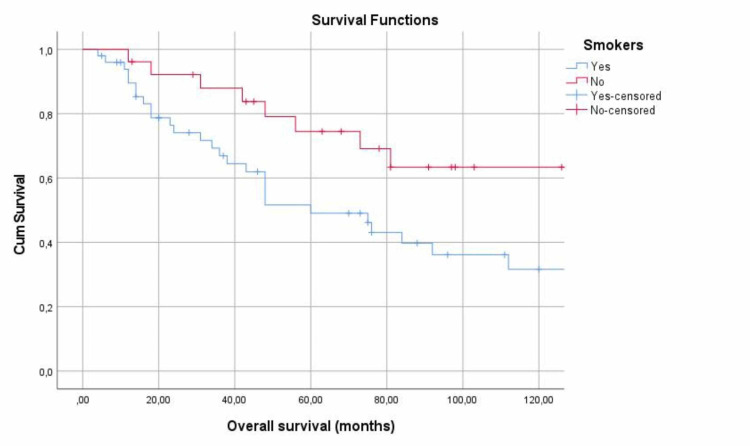
5- and 10-year survival in association to smoking habits

Smoking is one of the main risk factors for NPC and especially long-lasting (20 or more cigarettes a day). Statistical analysis showed that smokers had lower survival rates than non-smokers (p=0.016). Specifically, 49.1% of smokers were alive after 5-years and 31.6% after 10-years, whereas non-smokers showed higher survival rates of 74.5% and 63.4%, respectively (Figure 4).

Based on the cause of death (p=0.033), patients with metastatic or generalized carcinomatosis showed a 5- and 10-year survival rate of 32% and 12% and none of the relapsed patients was still alive after 5-years. In contrast with this, patients who died from other causes, unrelated to the disease, had a 50% survival rate after 5-years, but none of them survived 10-years after the diagnosis (Table 4).

**Figure 5:**
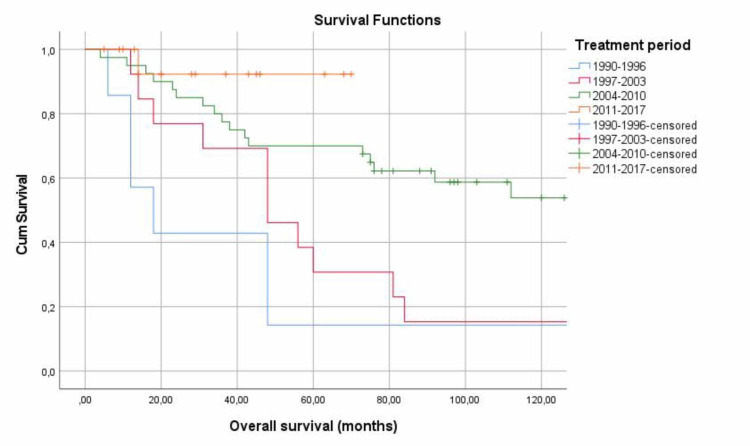
5- and 10-year survival in association to time period of diagnosis and treatment

Last but not least, we examined the survival relating to the time period when the diagnosis and treatment was done (p=0.002) (Table 4). Patients diagnosed and treated during the period 1990-1996 had the lowest 5- and 10-year survival rates (14.3% and 0%), while in the last period 2011-2017, we observed a 5-year survival rate of 92.3% (Figure 5).

## Discussion

Nasopharyngeal carcinoma (NPC) is the most common cancer originating from the nasopharynx, which occupies a separate geographical and tribal distribution [7]. The incidence of nasopharyngeal cancer in men is two to three times higher than in women [8]. Our study showed a higher incidence of diagnosis and cancer deaths in males, which is also shown in other studies [9]. Differences between men and women may be due to different lifestyle habits or biological indices [9,10].

In addition, in most low-risk groups, the incidence of NPC is stable with age increase [11,12]. We observed statistically higher 5-yearand 10-year survival rates in younger age groups compared to older age groups. Our findings are similar to those of other researchers [13], where a decrease in survival rates with age is reported. According to studies, this may be due to the fact that elderly patients at the time of diagnosis may die as a result of treatment complications, as they may be unable to tolerate the aggressiveness of the therapeutic approach [13].

Another factor studied was smoking, which is the most important risk factor for head and neck cancer. Over time, many studies [14,15] have highlighted that smoking is directly related to nasopharyngeal cancer and especially long-term heavy smoking [16]. In our study, over 50% of the incidents were active smokers. Statistical analysis showed that smokers had lower survival rates than non-smokers. The study by Rodriguez and Adelstein reports a negative impact on the survival of patients who continue to smoke after treatment, suggesting that smoking cessation can improve survival [17]. Also, according to Galbiatti et al. [18], there is a significant reduction in the risk of developing the disease when the patient stops smoking.

Currently, the mapping and prognosis of NPC patients are assessed primarily using the TNM Staging System. The clinical stage at diagnosis is an independent survival factor in both 5 and 10 years of follow-up. According to studies, the 5-year survival rate of patients with stage-I NPC was 90% or more [19]. Treatment of intermediate stage-II NPC results in 5-year survival and relapse-free survival of 77% and 62%, respectively, while about 30% of patients relapse with metastatic disease [20]. For stage-III and IV, the prognosis is poorer and relapse with distant metastases is common [21].

Lack of awareness continues to be a major barrier to the management of NPC in our area, which is added to delayed presentation to health services (64% of the cases were stage-III and IV). Regarding the survival analysis, we found higher survival rates in patients of stage-IV. Most of these patients were non-smokers, young, and received radiotherapy and chemotherapy. In contrast, literature data showing higher mortality rates in advanced stage-III and IV [22]. It is noteworthy that in some cases, there are differences between TNM stages and clinical outcomes [23]. Patients in the same staging class showed different survival effects due to the inability of the TNM system to reflect the biological heterogeneity between the tumors [7].

Nasopharyngeal carcinoma is traditionally treated with radical radiotherapy and with or without chemotherapy due to the radiosensitivity of the neoplasm and the difficulty in surgical access to it due to anatomical constraints [13,24]. In our study, almost all patients received radiotherapy with the exception of five cases that followed another treatment regimen. Moreover, NPC is a chemosensitive neoplasm, with rates of 60-74% responses to first-line chemotherapy [20]. According to bibliographic reports, no survival benefit from radiotherapy has been demonstrated, followed by adjuvant chemotherapy against radiotherapy alone, or by induction chemotherapy [25].

However, as shown in meta-analyses, the benefit of adding chemotherapy was mainly due to simultaneous administration with radiotherapy [26]. The results of our study are consistent with the above-mentioned reports, as we observed higher survival rates in patients receiving additional chemotherapy than those receiving radiotherapy only (5-year survival rate 23.3%).

Another important observation was the period of occurrence and treatment of the disease every seven years. We found that over time there were increased survival rates in these patients. Specifically, survival rates were found significantly higher during 2011-17 compared to 2004-10. Our findings complement other research that showed 5-year survival over the period 1971-2011 had increased for most cancers [27].

In addition, it is noteworthy to mention an epidemiological study from Taiwan showing a reduction by 30% in the age-standardized incidence rate between 1981 and 2000 [28].

The retrospective analysis of the last 28 years allowed us an insight into the survival rates of patients with NPC in Western Greece. Comparing the results of our study with those of other countries, no significant differences in survival rates are observed between Western Greece and European countries or the US [29-30], while large variations in the values of each parameter are seen in Malaysia, Singapore, Indonesia, Southeast Asia, Kenya in Northern Africa, and Southern China according to international literature [30].

However, the limitations should be taken into account when interpreting these results. First, retrospective analysis may inherently bias the study. Second, the relatively rare disease and the limited availability of complete records of the studied patients. Third, factors such as patients' blood groups, previous Epstein-Barr infection not studied. Finally, this study represents approximately 6.3% of the population of Greece.

## Conclusions

In conclusion, it is worth noting that a) a higher number of male than female patients were treated in the hospital, b) It is a rare form of cancer in Western Greece, c) Elderly patients in relation to younger age groups have a higher risk of relapse and lower overall survival, d) smoking seems to affect the 5-year and 10-year survival of these patients, e) 5-year and 10-year survival rates are influenced by age, diagnosis, and treatment choice, and f) the differences recorded in survival rates during the last two decades support that improvements in treatment and the introduction of the combined chemotherapy and radiotherapy have improved survival rates.
